# The point mutation A1387G in the 16S rRNA gene confers aminoglycoside resistance in *Campylobacter jejuni* and *Campylobacter coli*

**DOI:** 10.1128/aac.00833-24

**Published:** 2024-10-15

**Authors:** Michael Zarske, Christiane Werckenthin, Julia C. Golz, Kerstin Stingl

**Affiliations:** 1 German Federal Institute for Risk Assessment (BfR), Department of Biological Safety, National Reference Laboratory for *Campylobacter*, Berlin, Germany; 2 Lower Saxony State Office for Consumer Protection and Food Safety (LAVES), Food and Veterinary Institute, Oldenburg, Germany; University of Fribourg, Fribourg, Switzerland

**Keywords:** diagnostics, *Campylobacter*, antibiotic resistance, molecular genetics, aminoglycosides, DNA sequencing, food-borne pathogens

## Abstract

Thermotolerant *Campylobacter* spp. are the most frequent cause of foodborne bacterial diarrhea and high-priority antibiotic-resistant pathogens, according to the World Health Organization (WHO). Monitoring revealed current low prevalence of gentamicin resistance in European *Campylobacter* spp. isolates but substantial presence of gentamicin modifying genes circulating globally. Using a combined approach of natural transformation and whole-genome sequencing, we revealed a novel gentamicin resistance mechanism, namely the point mutation A1387G in the 16S rRNA gene, originally identified in a *C. coli* isolate from turkey caecal content. The transformation rate of the resistance using genomic DNA of the resistant donor to sensitive recipient *C. jejuni* and *C. coli* was ~2.5 log_10_ lower compared to the control *rpsL*-A128G point mutation conferring streptomycin resistance. Antimicrobial susceptibility tests showed cross-resistance to apramycin, kanamycin, and tobramycin, with transformants exhibiting more than 4- to 8-fold increased MICs to apramycin and tobramycin and over 64-fold higher MICs to kanamycin compared to wild-type isolates. Although transformants showed 177–1,235 variations relative to the recipient, only the A1387G point mutation in the 16S rRNA was in common. This mutation was causal for resistance, as transformation of a 16S rRNA_A1387G PCR fragment into susceptible isolates also led to resistant transformants. Sanger sequencing of the 16S rRNA genes and Oxford nanopore whole-genome sequencing of transformants identified clones harboring either all three copies with A1387G or a mixed population of wild-type and mutated 16S rRNA gene alleles. Within 15 passages on non-selective medium, transformants with mixed populations of the 16S rRNA gene copies partially reverted to wild type, both geno- and phenotypically. In contrast, transformants harboring the A1387G point mutation in all three 16S rRNA gene copies kept full resistance within at least 45 passages. We speculate that partial acquisition and rapid loss of the point mutation limited its spread among *C*. spp. isolates. In-depth knowledge on resistance mechanisms contributes to optimal diagnosis and preventative measures.

## INTRODUCTION

In 2022, the leading cause of bacterial gastroenteritis in the European Union was again *Campylobacter* spp. with 137,000 reported Campylobacteriosis cases, that is, more than twice the number of reported *Salmonella* infections (1). Patients with acute campylobacteriosis show symptoms like watery and/or bloody diarrhea, abdominal cramps, fever, and nausea (2). Furthermore, there is a potential for the development of long-term autoimmune sequelae following an acute infection, such as Guillain-Barré syndrome, reactive arthritis, and irritable bowel syndrome (3). While antibiotic therapy may not be required for the majority of cases of food-borne campylobacteriosis, patients with severe or persistent infections necessitate antimicrobial treatment (4). Fluoroquinolones and macrolides are the preferred pharmaceutical agents applied in clinics for the treatment of campylobacteriosis (5, 6). However, globally emerging antimicrobial resistances (AMRs) are impeding the effectiveness in treatment with these agents, in particular for (fluoro-)quinolones (7–9). Thus, in cases of systemic infection, aminoglycoside antibiotics, specifically gentamicin, persist as the recommended therapeutic option given the susceptibility of *Campylobacter* isolates to this particular class of antibiotics (10).

Aminoglycosides are a class of potent broad-spectrum antibiotics derived from actinomycetes, which have been in use since the 1940s (11). Their primary mode of action involves inhibition of bacterial protein synthesis via blocking elongation or directly inhibiting initiation, with the exact mechanism varying by chemical structure (12–15). Aminoglycosides can be subdivided into two main classes based on the core structure of the aminocyclitol moiety: those derived from streptidine (e.g., streptomycin) and those derived from 2-deoxystreptamine (e.g., gentamicin and kanamycin). The 2-deoxystreptamine-derived aminoglycosides further divide into subclasses based on the specific substitution pattern of their side chains. These structural differences are crucial for their mechanisms of action and susceptibility to various aminoglycoside-modifying enzymes (16). Resistance to gentamicin is rare in Europe (17, 18), but more frequently encountered in China (19, 20), Vietnam (21), and the Philippines (22). Aminoglycoside resistance in *Campylobacter* spp. is mainly attributed to enzymatic inactivation of the aminoglycoside by enzymes. These enzymes include aminoglycoside *N*-acetyltransferases (AAC), *O*-phosphotransferases (APH), and *O*-nucleotidyltransferases (ANT), which are the predominant mechanisms of resistance (23–25). Resistance to gentamicin, kanamycin and tobramycin is conveyed by the presence of aminoglycoside 2″-phosphotransferase genes [*aph(2''*)] with several distinct variants identified in *Campylobacter* (19, 26–31). Kanamycin resistance is also attributed to the presence of 3′-phosphotransferase genes, such as *aph(3')-IIIa* (32) and *aph(3')-VIIa* (33). A recent study found the presence of the resistance gene *apmA* in a *C. coli* isolate which may encode an acetyltransferase for inactivating apramycin (30).

In this study, we report the identification of a novel point mutation in the bacterial A-site of the 16S rRNA in a German *C. coli* isolate that causes resistance to aminoglycosides with a 2-deoxystreptamine structure. We investigated the transferability of this mutation among thermotolerant *Campylobacter* spp. through natural transformation and evaluated its stability under non-selective conditions. Our results highlight the importance of in-depth investigation of the mechanisms of antimicrobial resistance in food-borne pathogens in order to evaluate their spread and persistence. This will improve prediction of resistances using current diagnostics of whole-genome sequencing.

## RESULTS

### Identification of a *C. coli* isolate from turkey caecum with an unknown gentamicin resistance mechanism

Routine resistance monitoring of thermotolerant *Campylobacter* spp. isolated from German turkey in 2018 revealed the presence of a *C. coli* isolate (BfR-CA-15687) displaying gentamicin resistance with a minimum inhibitory concentration (MIC) value >16 mg/L (21). This isolate displayed ST type 10049, which is not yet assigned to a clonal complex. The antimicrobial microdilution assay using the EU-wide harmonized EUCAMP3 plate format was conducted in three independent experiments, all confirming high-level gentamicin resistance. In addition, the isolate was resistant to ciprofloxacin and tetracycline (Table 1; Fig. S1A). Whole-genome sequencing based on Illumina short-reads was carried out to identify known resistance determinants. However, no known resistance determinant associated with gentamicin resistance was detected in the assembly or in trimmed reads. As expected for ciprofloxacin and tetracycline-resistant *C*. spp., the presence of the T86I point mutation in gyrase subunit A and two copies of the *tet*(O) resistance gene—one located on the chromosome and the other on the plasmid—were detected. The isolate additionally harbored a *bla*_OXA-489_ gene with a G to T transversion at position −57 in the promoter region, expected to restore the Pribnow box and to confer resistance to ampicillin (34), which was not phenotypically characterized. The short-read sequencing was repeated, again leading to lack of any known resistance determinants for the observed gentamicin resistance.

**TABLE 1:** MIC values of BfR-CA-15687, including test ranges and resistance evaluation^*a*^

Antimicrobial	Plate format	Test range (mg/L)	Resistant > MIC (mg/L)	MIC of BfR-CA-15687	Evaluation (R, S)
Apramycin	Custom	0.03–32	16^*b*^	>32	R
Chloramphenicol	EUCAMP3	2–64	16	4	S
Ciprofloxacin	EUCAMP3	0.12–32	0.5	32	R
Ertapenem	EUCAMP3	0.12–4	0.5	0.12	S
Erythromycin	EUCAMP3	1–512	4 (Cj), 8 (Cc)	2	S
Gentamicin	EUCAMP3	0.25–16	2	>16	R
Kanamycin	Custom	1–1024	16^*b*^	>1,024	R
Streptomycin	Custom	0.25–16	4	1	S
Tetracycline	EUCAMP3	0.5–64	1 (Cj), 2 (Cc)	>64	R
Tobramycin	Custom	0.06–64	16^*b*^	>64	R

^*a*^ For resistance evaluation, epidemiological cutoff values (ECOFF) were used except where indicated. R, resistant; S, sensitive; MIC, minimal inhibitory concentration; Cj, *C. jejuni*; Cc, *C. coli*.

^*b*^ For resistance evaluation, elevated non-wild-type MICs were used.

The known gentamicin phosphorylating Aph(2″) enzyme variants not only confer resistance to gentamicin (GEN) but also to the aminoglycosides kanamycin (KAN) and tobramycin (TOB) (21, 25). Hence, BfR-CA-15687 was also tested for susceptibility to these two aminoglycosides as well as to apramycin (APR) and streptomycin (STR) using custom-created plates (Table 1; Fig. S1B and C). BfR-CA-15687 additionally demonstrated elevated non-wild-type MIC values for APR (>32 mg/L), KAN (>1,024 mg/L), and TOB (>64 mg/L). In comparison, the tested wild-type isolates (*C. jejuni* BfR-CA-14430, *C. coli* BfR-CA-14856) and reference strains (81-176, NCTC 11168) displayed sensitivity to GEN and notably lower MIC values for the aminoglycosides APR (4–8 mg/L), KAN (8–16 mg/L), and TOB (4–8 mg/L) (Table 2). Given the absence of determinants linked to APR, KAN, or TOB resistance, these results indicated that the unidentified gentamicin resistance determinant might be a potential factor also contributing to the observed elevated MIC values to APR, KAN, and TOB.

**TABLE 2:** MIC values of the donor *C. coli* BfR-CA-15687, wild-type recipient isolates, and transformant strains for aminoglycosides^*a*^

Strain	Species	Sample	MIC [mg/L]				
			APR	GEN	KAN	STR	TOB
Donor isolate	*C. coli*	BfR-CA-15687	>32	>16	>1,024	1	>64
Wild-type recipient isolates	*C. coli*	BfR-CA-11057	4	0.5	16	1	8
		BfR-CA-14856	8	0.5	16	1	8
	*C. jejuni*	BfR-CA-14430	4	0.5	8	1	4
		NCTC 11168	4	0.25	8	0.5	8
		81–176	4	0.25	8	1	8
TF using gDNA_BfR-CA-15687_	*C. coli*	BfR-CA-11057-TF15687	>32	>16	>1,024	1	>64
		BfR-CA-14856-TF15687	>32	>16	>1,024	2	>64
	*C. jejuni*	81-176-TF15687	>32	>16	>1,024	1	>64
TF using 16SrRNA fragment_BfR-CA-15687_	*C. coli*	BfR-CA-11057-TF16S	>32	>16	>1,024	1	>64
		BfR-CA-14856-TF16S	>32	>16	>1,024	2	>64
	*C. jejuni*	BfR-CA-14430-TF16S	>32	>16	>1,024	0.5	>64
		NCTC 11168-TF16S	>32	>16	>1,024	1	>64
		81-176-TF16S	>32	>16	>1,024	1	>64

^*a*^ MIC, minimal inhibitory concentration; TF, transformant; TF16S, transformant after transformation of the 16S rRNA PCR fragment; APR, apramycin, GEN, gentamicin, KAN, kanamycin, STR, streptomycin, TOB, tobramycin.

### Natural transformation experiments showed that the unknown APR-GEN-KAN-TOB resistance is transferable among isolates

To explore the transferability of the observed APR-GEN-KAN-TOB resistance through natural transformation, genomic DNA from isolate BfR-CA-15687 was used to naturally transform different recipient strains, which were sensitive to gentamicin and displayed low MICs for apramycin, kanamycin and tobramycin (Table 2). The *C. coli* and *C. jejuni* transformants were selected on 16 and 8 mg/L TOB, respectively, in order to provide a selective pressure at the MIC or maximally twofold MIC of the respective recipient strain. As a control, genomic DNA from a streptomycin-resistant *Campylobacter jejuni* transformant BfR-CA-14430-strep, harboring the *rpsL*_A128G_ point mutation, was used and transformants were selected on 16 mg/L STR (35). This allowed for the quantification of natural transformation capacity and normalization of the transformation rate.

**Fig 1:**
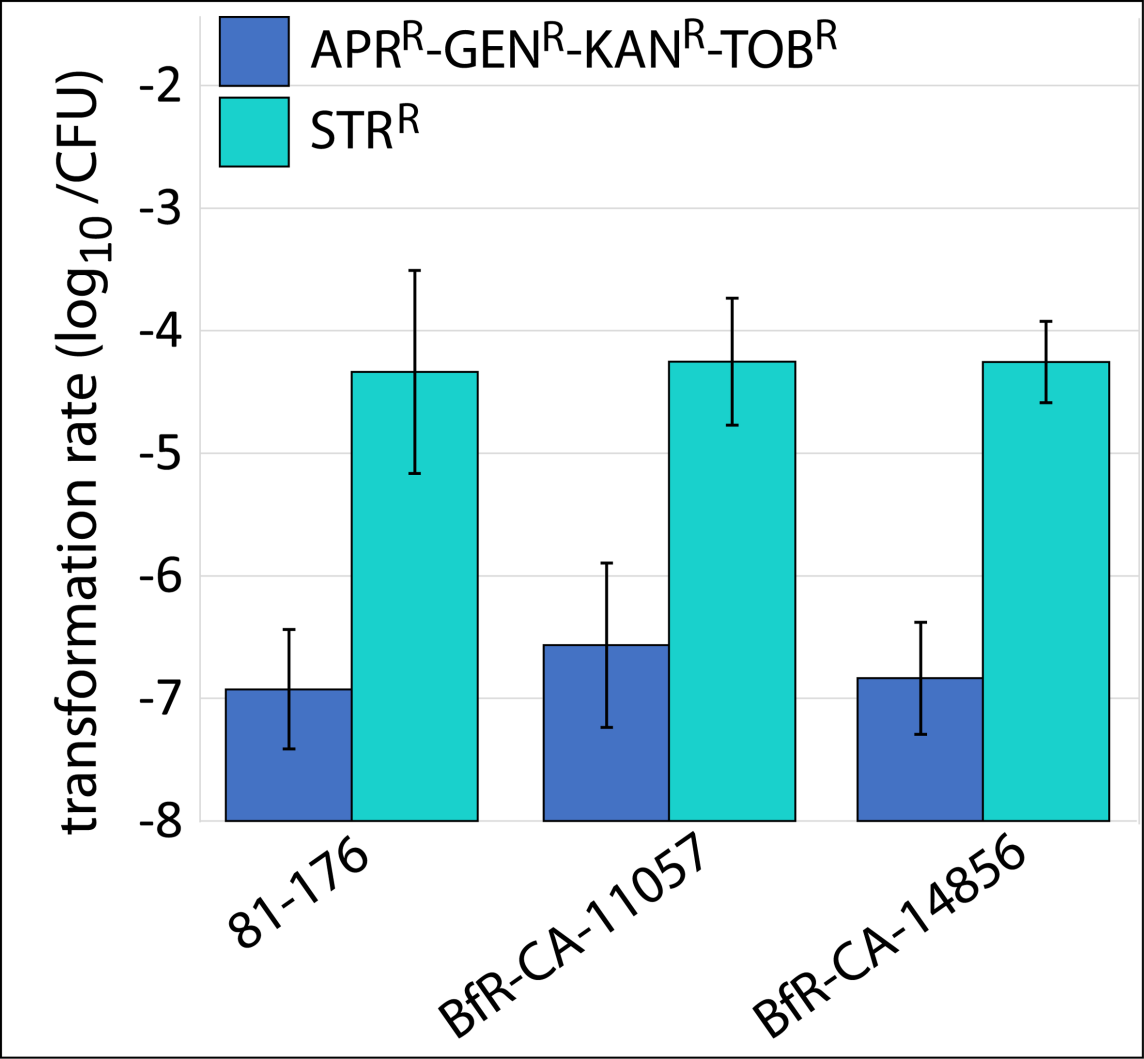
The transformation rates of APR^R^-GEN^R^-KAN^R^-TOB^R^ resistance determinant using gDNA of BfR-CA-15687 was ~2.5 log_10_ lower (blue bars) than transformation of the control *rpsL*_A128G_ point mutation using gDNA of BfR-CA-14430-strep leading to STR^R^ (turquoise bars). The sensitive wild-type strains *C. jejuni* 81-176, *C. coli* BfR-CA-11057, and *C. coli* BfR-CA-14856 were transformed with 1 µg/mL gDNA. Transformation rates were assessed from the ratio of resistant transformants relative to CFU on non-selective Columbia blood agar. The data stem from at least three independent experiments, with error bars representing standard deviation.

When transforming gDNA of BfR-CA-15687 into recipient strains *C. jejuni* 81-176, *C. coli* BfR-CA-11057, and *C. coli* BfR-CA-14856, we observed transformation rates of approximately 10^−7^ per CFU after 48 hours of incubation (Fig. 1). As control, transformation of the *rpsL*_A128G_ point mutation in the same recipient strains was around 2.5 log_10_ more efficient, with transformation rates ranging from 2.23 × 10^−5^ to 1.26 × 10^−4^ per CFU (Fig. 1). Additionally, when using a PCR fragment of the 16S rRNA_A1387G as substrate, transformation rates were in mean 4.51 × 10^−6^ ± 1.48 × 10^−6^ per CFU (overall mean of the strains, *n* = 5).

### Whole-genome sequencing analysis elucidates a correlation between aminoglycoside resistance and a novel 16S rRNA gene mutation

**Fig 2:**
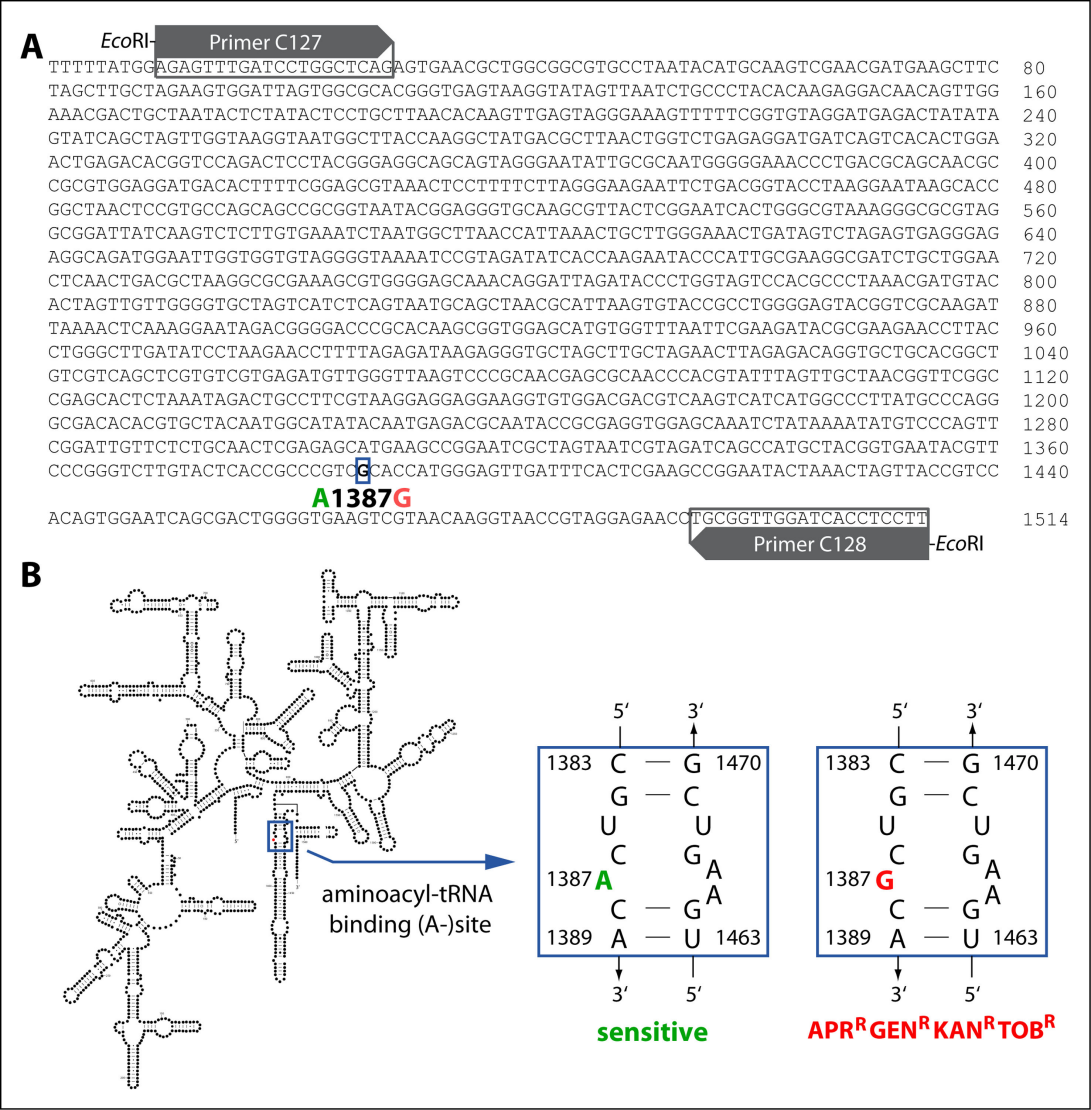
Primary sequence of the 16S rRNA of *C. coli* BfR-CA-15687 (A) and proposed secondary structure of *Campylobacter* spp. 16S rRNA [(B), modified from reference (36)] with the location of the point mutation highlighted with blue frames in (A and B), leading to APR^R^-GEN^R^-KAN^R^-TOB^R^ resistance phenotype. Blue frame in (B), sequence of the aminoacyl tRNA decoding region (A-site) of sensitive (1387A in green) and resistant (1387G in red) phenotypes. Numbers indicate positions in the *C*. spp. 16S rRNA sequence. Forward and reverse primers (gray boxes) flanked with 5′-*Eco*RI motifs are depicted in (A), which were used for amplification of a 16S rRNA gene fragment of BfR-CA-15687, transformed into sensitive recipient strains.

In order to gain insight into the genetic determinant of the APR-GEN-KAN-TOB resistance in BfR-CA-15687, whole-genome sequencing of the sensitive BfR-CA-11057 recipient and nine of its isogenic APR-GEN-KAN-TOB resistant transformants derived from two independent transformation experiments using gDNA of BfR-CA-15687 were compared. For this purpose, the donor isolate BfR-CA-15687 and the recipient isolate BfR-CA-11057 also underwent Oxford Nanopore long-read sequencing in addition to short-read sequencing to obtain a closed genome upon Unicycler hybrid assembly. Mapping of trimmed reads derived from each transformant to the hybrid assembly of BfR-CA-11057 revealed 177–1,235 sequence nucleotide variants (SNPs) relative to the recipient strain (Table S1B). “Unused reads,” which did not map to the recipient, were subsequently mapped to the donor strain BfR-CA-15687 hybrid assembly sequence. However, those reads did not map to any sequence region common to all transformants (Table S2). Moreover, the transformants exhibited no similarities among genetic regions with low coverage, putatively representing deleted regions compared to the recipient (Table S3). Intriguingly, the consensus of all examined variations in transformants relative to the recipient was a mutation in all three copies of the 16S rRNA gene, located in the aminoacyl tRNA decoding A-site (A1387G; *E. coli* numbering A1408G; Table S1A and B; Fig. 2), which was also present in the donor strain BfR-CA-15687 (NCBI; genome accession CP126367-CP126368, BioSample accession SAMN34728731).

### *In vitro* transformation experiments using a PCR fragment of the 16S rRNA of BfR-CA-15687 verified A1387G point mutation in 16S rRNA gene as a novel mechanism for aminoglycoside resistance in *C. jejuni* and *C. coli*

In order to ascertain that the phenotypic resistance was indeed solely attributed to this novel point mutation, we amplified the majority of the 16S rRNA gene (10–1,514 bp) of BfR-CA-15687 by PCR (Fig. 2A). In order to render the PCR fragment mobilizable via natural transformation in *C. jejuni* and *C. coli*, a 5′-*Eco*RI motif was introduced at both ends of the PCR fragment and the fragment was methylated by an *Eco*RI methylase before use as DNA substrate in the natural transformation experiments (35, 37).

For all five aminoglycoside sensitive recipient strains, APR-GEN-KAN-TOB resistant transformants were obtained by transformation of the 16S rRNA PCR fragment of BfR-CA-15687 (Table 2). Subsequent short-read sequencing unveiled the A1387G mutation within the 16S rRNA gene of all five analyzed transformants (one per parental strain), providing corroborative evidence for the causal association between this specific mutation in the 16S rRNA gene and phenotypic resistance to APR-GEN-KAN-TOB in *C. jejuni* and *C. coli*.

We investigated whether the 16S rRNA gene point mutation could be found in previously published sequences of *C. coli*. Using the Basic Local Alignment Search Tool (BLAST) at NCBI, we screened over 32,000 publicly available *C. coli* whole-genome sequences. However, we did not find any instance of this mutation.

### Transformants harbor different numbers of 16S rRNA_A1387G gene copies per chromosome

**Fig 3:**
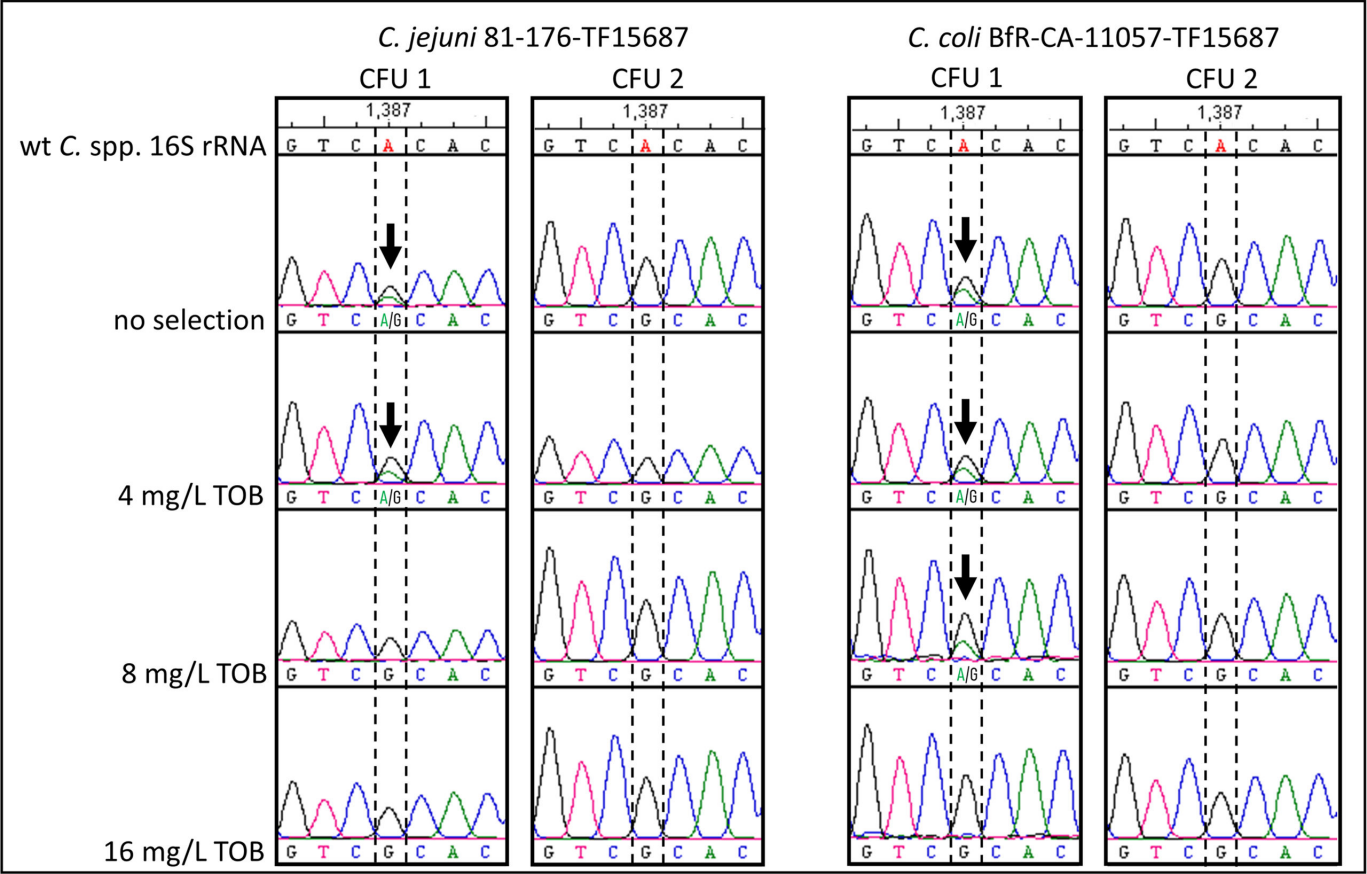
Single colonies of transformants switched from a mixed A/G genotype at position 1387 in 16S rRNA to only G at higher TOB concentrations. Two representative transformant colonies (CFU 1 and CFU 2) of each *C. jejuni* 81–176-TF15687 and *C. coli* BfR-CA-11057-TF15687 after transformation were transferred to different concentrations of TOB and in parallel on non-selective ColbA. Sanger sequencing revealed two populations of resistant transformants—either harboring base G upon transformation or a mixture of bases A and G at position 1387 in the 16S rRNA genes (marked with black arrows), which changed to only G at higher TOB concentrations. The base color code of the Sanger sequences is indicated below the chromatograms. TOB, tobramycin; wt, wild-type.

We intended to know, whether all three 16S rRNA gene copies, present in *C. jejuni* and *C. coli* chromosomes, displayed the A1387G point mutation in the transformants, leading to TOB resistance. Hence, just after natural transformation with gDNA, single TOB-resistant transformant colonies of BfR-CA-11057-TF15687 and 81-176-TF15687 were once subcultured on ColbA. Subsequently, DNA was extracted from this first passage and the 16S rRNA fragment comprising position 1387 was Sanger sequenced. We observed two different genotypes of transformants on non-selective ColbA—either a G was detected at position 1387 (Fig. 3, CFU 2, no selection) or a double peak in the chromatogram of the Sanger sequence, corresponding to a mixed population of A and G at position 1387 was identified (Fig. 3, CFU 1, no selection).

Furthermore, single colonies just after transformation were streaked on plates with varying concentrations of TOB (4, 8, and 16 mg/L) and without TOB supplementation (Fig. 3). The colonies from non-selective plates with only G at position 1387 maintained this genotype independent of the TOB concentration the colony was characterized from. However, colonies from non-selective plates, showing a mixed population of A and G at position 1387 in the 16S rRNA gene, switched to a “pure” G at position 1387 at either 8 or 16 mg/L TOB (Fig. 3). Hence, the relative ratio of transformants with a distinct number of 16S rRNA gene copies with A1387G appeared to be dynamic, unless all three copies displayed the resistance determining mutation.

We further wanted to decipher, whether the mixed A/G population of 16S rRNA gene sequences at position 1387 was caused by a mixture of 16S rRNA gene variants in the same bacterium. For this purpose, Oxford Nanopore Technologies (ONT) sequencing was performed on a freshly obtained transformant of 81–176-TF15687 only once passaged on non-selective ColbA and displaying a mixed population of A/G at position 1387 in the Sanger sequence. The same DNA was subjected to Illumina short-read sequencing. Using long-read and short-read sequences a hybrid assembly was created using Unicycler, leading to a closed chromosome and one plasmid of 44.8 kb. To rule out assembly errors, the trimmed long-reads of the 81–176-TF15687 transformant were mapped to its hybrid assembly. All three copies of the 16S rRNA gene located in the three ribosomal RNA (rrn) operons at positions 39,157–44,986 bp (rrn I), 395,917–401,610 bp (rrn II), and 693,077–698,770 bp (rrn III) of the chromosome were visualized (Fig. S2). Indeed, in this transformant, the 16S rRNA gene at rrn I and rrn II displayed the A1387G point mutation, whereas the copy at position rrn III maintained wild-type base A at position 1387.

### The copy number of 16S rRNA genes with A1387G is important for persistence of aminoglycoside resistance

**Fig 4:**
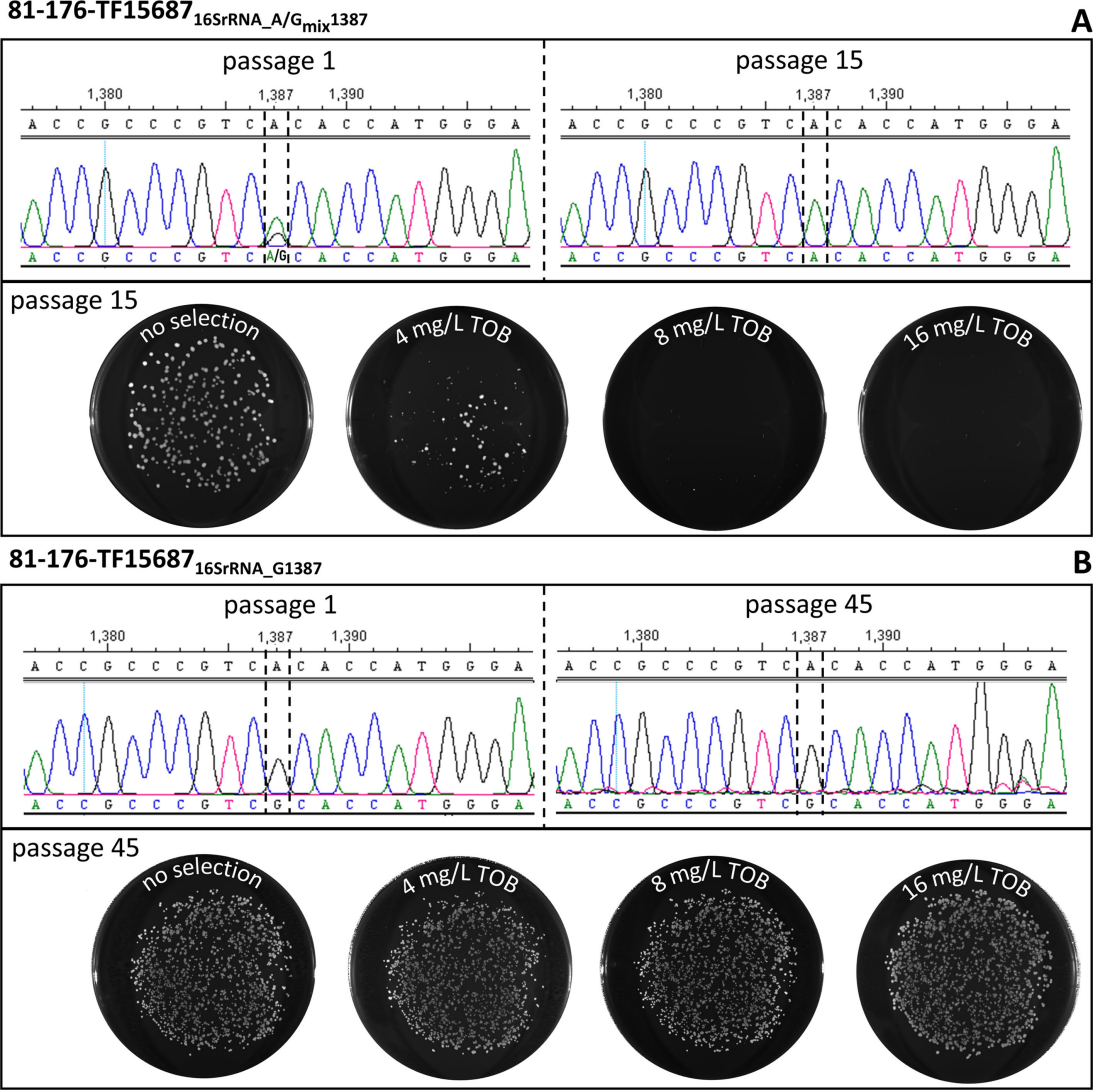
A transformant harboring a mixed A/G genotype at position 1387 in 16S rRNA reverted to a sensitive phenotype after 15 passages (**A**). In contrast, a transformant with only G at position 1387 maintained resistance even after 45 passages (**B**). Transformants of *C. jejuni* 81-176-TF15687 were passaged on non-selective ColbA. After the indicated number of passages, the transformant culture was diluted and spread on non-selective ColbA plates in order to obtain single colonies. Subsequently, colony material was transferred on plates with different concentrations of TOB by stamping with velvet cloth. Photographs of colony patterns on each plate were captured after the indicated number of passages. Sanger sequences are shown after passage 1 (fresh transformant) and after repeated subculturing. After passaging, a colony was taken from non-selective ColbA for Sanger sequencing.

Furthermore, we intended to evaluate the stability of the newly acquired resistance. For this purpose, fresh transformant colonies of 81-176-TF15687 were first serially diluted to form new single colonies. Subsequently, representative colonies were repeatedly subcultured on non-selective ColbA. Upon passage 1, the genotype at position 1387 in the 16S rRNA genes was initially analyzed by Sanger sequencing (Fig. 4). As mentioned above, either a mixed A/G genotype at position 1387 in 16S rRNA or only G at this position was observed (81-176-TF15687_16SrRNA_A/Gmix1387_ or 81-176-TF15687_16SrRNA_G1387_, respectively). After 15 passages on non-selective ColbA, the resistance to TOB was reassessed in transformants by agar dilution, which had initially been shown to harbor the mixed A/G genotype. Colonies of the subcultured transformants, that were stamped, i. e. transferred by velvet cloth on ColbA plates with different concentrations of TOB, revealed loss of resistance to 8 and 16 mg/L TOB after passaging. Thus, colonies were only observed on non-selective medium and, in a smaller quantity, in the presence of 4 mg/L TOB (Fig. 4A). The reversion to a sensitive genotype (only A at position 1387 in the 16S rRNA gene) was confirmed by Sanger sequencing for a colony, which only grew on non-selective ColbA (Fig. 4A). After passaging 81-176-TF15687_16SrRNA_G1387_ for 45 times, which initially only displayed G at position 1387, the phenotypic assay revealed stability of resistance to TOB. In particular, the number of colonies with and without TOB was stable, independent on the concentration of TOB (Fig. 4B). Here, Sanger sequencing of a representative colony showed that the G base at position 1387 was still the only base present after passaging, thus, suggesting stable G1387 presence in all three copies of the 16S rRNA gene.

## DISCUSSION

According to regular zoonosis surveillance of thermotolerant foodborne *Campylobacter* spp., resistance to gentamicin remains rare in Europe (17). In 2022, gentamicin resistance was observed in 2% of the *C. coli* isolates and 0.1% of *C. jejuni* isolates from broiler, and in 3% of *C. coli* and 0.5% of *C. jejuni* isolates from infected humans. Thus, this antibiotic substance can still be considered effective for treatment of campylobacteriosis. However, in China, 15.6% of *C. jejuni* and 79.9% of *C. coli* isolates from chicken and swine collected in 2014 and 13% of *C. jejuni* as well as 50% of *C. coli* isolated from humans in 2017–2018 showed gentamicin resistance (19, 20). Likewise, a high proportion of *C*. spp. isolates from chicken were gentamicin resistant in Vietnam [21.9% and 78.8% in *C. jejuni* and *C. coli* isolated from broiler, respectively (21)] and in the Philippines [65.2% (22)]. Although point mutations in the 16S rRNA gene have been linked with 2-deoxystreptamine aminoglycoside resistance in other organisms (38–41), this has never been observed before in *C*. spp.. In this study, we have demonstrated that the A1387G mutation occurring at the bacterial A-site (aminoacyl tRNA binding site; Fig. 2) confers resistance to the aminoglycosides APR, GEN, KAN, and TOB in *C. coli* and *C. jejuni*. Target site modifications, involving genetic mutations and enzymatic methylation predominantly occur at the bacterial A-site, which serves as the binding site for most aminoglycosides (37, 42, 43).

The aminoglycosides used in this study, except streptomycin, all have a 2-deoxystreptamine backbone in common. While GEN, KAN, and TOB belong to the 4,6-disubstituted class with an ammonium group (NH_3_^+^) at position 6′ of ring I, APR differs in its structural appearance by belonging to the 4-monosubstituted subclass, while having a hydroxy group (OH) at position 6′ of ring I. Nevertheless, these four aminoglycosides have rings I and II in common and either residue (NH_3_^+^, OH) of ring I is able to build a Watson-Crick pseudo pair with 16S rRNA A1408 (numbering in *E. coli*, corresponding to A1387 in *C*. spp.), thereby inhibiting the decoding step of protein biosynthesis of the bacterium (16).

We wondered, why we were the first to describe this point mutation in *Campylobacter* and if there were sequences published that harbor this specific 16S rRNA gene point mutation. Thus, we conducted analyses utilizing the NCBI BLAST tool and screened the database of whole-genome sequences publicly available. However, we could not find the point mutation in any of the more than 32,000 whole-genome sequencing data sets of *C. coli*, suggesting that the resistance determinant identified in the field isolated from caecum of a turkey in Germany during routine monitoring is rare. Likewise, its transferability via horizontal gene transfer was inefficient, with a ~2.5 log_10_ lower transformation rate compared to the control *rpsL*_A128G_ point mutation. In addition, we only observed transformants when using relatively low initial concentrations of selective aminoglycoside, just above the MIC of the respective wild-type recipients. If transformant colonies were picked initially and streaked on increasing concentrations of selective aminoglycoside, we observed adaptation of colonies with initial mixed 16S rRNA gene copies with and without A1387G mutation to only G at position 1387 at higher concentrations (Fig. 3). This likely stems from a gradual transition from a sensitive to a fully resistant phenotype. Hence, we concluded that colonies that have undergone only partial transition are still impeded in growth due to the selective pressure at higher concentrations of aminoglycoside, potentially resulting in a reduced growth rate. Likewise, loss of resistance was observed upon few passages on non-selective medium (Fig. 4A), while the resistance was stable if all three copies of the 16S rRNA genes harbored G at position 1387 (Fig. 4B). The combined results suggest that complete resistance is only evident when all three copies of the 16S rRNA gene have acquired the A1387G point mutation but that acquisition might be the limiting factor. Interestingly, previous studies reported that thermotolerant *C*. spp. carry aminoglycoside modifying enzymes, such as Aph(2″) phosphotransferase variants (19, 28, 29). Thus, we speculate that although the presence of all three copies of the 16S rRNA gene provides a high level of gentamicin resistance in *C*. spp., the gradual acquisition of mutated 16S rRNA gene(s) with a low level of resistance may not provide sufficient advantage under selection pressure. In Germany, gentamicin resistance in *C*. spp. is very low, with the isolation of only single isolates per year during zoonosis monitoring. From our analysis, it is tentative to speculate that the 16S rRNA_A1387G gene in the *C. coli* from caecal content of turkey may have emerged by sublethal concentrations of selective agent over time, which remains to be further investigated for potential future practical consequences. A similar phenomenon of gradual acquisition of point mutations in multiple copies of the 16S rRNA gene was observed in *Nocardia farcinica* exposed to amikacin for 24 h (39). Here, the three 16S rRNA genes showed increasing copy number containing the A1408G point mutation, leading to all copies with a G at position 1408 after prolonged incubation under selective conditions.

In the absence of selective pressure, we did not detect any growth deficiencies on blood agar in transformants, which stably acquired G in all copies of the 16S rRNA gene. However, easy loss of the point mutation upon passage on non-selective blood agar in transformants with mixed copies of A and G at position 1387 indicated fitness costs *in vitro* in *C*. spp. In other bacterial pathogens, like *Mycobacteria* (42, 44) and *Borrelia burgdorferi* (43), the point mutation in the 16S rRNA gene is frequently found as resistance determinant for gentamicin in clinical isolates, demonstrating principle toleration and full function of the mutated 16S rRNA gene *in vivo* at least in some bacterial species.

Macrolide resistance in *Campylobacter* is primarily attributed to the A2075G point mutation in the 23S rRNA gene (45). In contrast to the 16S rRNA_A1387G gene mutation, the macrolide resistance conferring mutation was reported to be stable upon passaging on non-selective medium, even in isolates harboring only two out of three copies of the 23S rRNA gene with A2075G (46). This enhanced stability might be a factor contributing to its frequent occurrence in macrolide-resistant *Campylobacter*, while antibiotic-resistant mutations in the 16S rRNA gene have not been observed before. However, also the macrolide conveying 23S rRNA mutations have been shown to lead to fitness costs in *C. jejuni* in chicken (47). Nevertheless, further investigations are required to evaluate the potential loss of fitness of the 16S rRNA_A1387G gene mutation also *in vivo*. Here, strains with all three 16S rRNA gene copies harboring the A1387G mutation might be tested over time for colonization capacity in a chicken model. In particular, it would be interesting to challenge the resistant strain in competition with an isogenic strain, carrying all three wild-type 16S rRNA gene copies with A1387.

### Conclusion

The novel point mutation A1387G in the 16S rRNA gene of *C. jejuni* and *C. coli* was revealed as novel causative determinant for APR, GEN, KAN, and TOB resistance. However, acquisition in less than all three copies of the three 16S rRNA genes in *C*. spp. led to rapid loss and return to a sensitive phenotype. This phenomenon putatively contributed to *C. coli* BfR-CA-15687 being the first and to our knowledge yet only isolate, harboring this resistance. Understanding the molecular mechanism of resistance as well as their acquisition and persistence in pathogens is crucial for combatting the spread of resistance globally.

## MATERIALS AND METHODS

### Strains and growth conditions

*C. coli* BfR-CA-11057, BfR-CA-14856 and BfR-CA-15687 were isolated in Germany in the years 2012, 2016, and 2018 from raw cow milk, raw goat milk, and caecum of turkey, respectively. *C. jejuni* isolate BfR-CA-14430 was obtained from fresh chicken meat in Germany in 2016 (48). Isolation was conducted by the Federal State Laboratories according to EN ISO 10272-1 valid in the respective year (49, 50). In addition, reference strains *C. jejuni* NCTC 11168 (51), 81-176 (52), and DSM 4688 (DSMZ—German Collection of Microorganisms and Cell Cultures GmbH, Braunschweig, Germany) as well as *C. coli* strain 2012-70-443-2 (Technical University of Denmark, Lyngby, Denmark) were used. If not stated otherwise, incubation of all cultures was performed under microaerobic conditions with 5% O_2_, 10% CO_2_, and 85% N_2_ (Binder, Tuttlingen, Germany). Cultures derived from −80°C stock cultures (MAST Group Ltd., Bootle, UK) were cultivated on Columbia blood agar plates containing 5% defibrinated sheep blood (ColbA, Oxoid, Thermo Fisher Scientific Inc., Waltham, MA, USA) at a temperature of 42°C for 24 h. Subsequently, the bacteria were subcultured on ColbA for 20 ± 2 h before use. For the selection of transformants, ColbA was supplemented with either tobramycin (8–16 mg/L) or streptomycin (16 mg/L) (Sigma Aldrich, St. Louis, MO, USA, respectively).

### PCR amplification and methylation of 16S rRNA

For the amplification of the 16S rRNA of target sequence BfR-CA-15687, forward primer C127 (5′-CTA GCG AAT TCA GAG TTT GAT CCT GGC TCA G-3′) and reverse primer C128 (5′-GGA CTG AAT TCA AGG AGG TGA TCC AAC CGC A-3′) were used, carrying each an *Eco*RI motif at the 5′ end. The PCR amplification was performed using Q5 High Fidelity DNA Polymerase (New England Biolabs, Ipswich, MA, USA), following PCR fragment purification using the QIAquick PCR Purification Kit (Qiagen, N.V., Venlo, The Netherlands). Subsequently, the PCR fragment was methylated using an *Eco*RI methyltransferase (New England Biolabs, Ipswich, MA, USA) for 1 h at 37°C, followed by heat inactivation at 65°C for 15 min. This procedure is mandatory for mobilization of the PCR fragment for DNA uptake by *C*. spp. (37, 53).

### Sanger sequencing analysis of 16S rRNA genes

The primers 16SrRNA-F1 (5′-AGA GTT TGA TCC TGG CTG AG-3′) and 16SrRNA-R1 (5′-AAG GAG GTG ATC CAG CCG CA-3′) (54) were used for amplification applying the Q5 High Fidelity DNA Polymerase (New England Biolabs, Ipswich, MA, USA). The PCR fragment was purified using the QIAquick PCR Purification Kit (Qiagen, N.V., Venlo, The Netherlands) and 1.5 µg of the DNA was supplemented with the sequencing primer 16SrRNA-S4 (5′-AGT CCC GCA ACG AGC GCA AC-3′) (55) for Sanger sequencing at Eurofins Scientific SE, Luxembourg City, Luxembourg.

### DNA uptake assay and transformation

Recipient strains from a 20 h ± 2 h preculture on ColbA were resuspended in 5 mL of brain heart infusion (BHI, Oxoid, Thermo Fisher Scientific Inc., Waltham, MA, USA) and adjusted to an optical density (OD) at 600 nm of 0.3. Subsequently, the strains were cultured at 140 rpm and 37°C in an atmosphere containing 3.5% H_2_, 6% O_2_, 7% CO_2_, and rest N_2_ for 6 h. Cultures were passaged to fresh BHI and grown over night at the same conditions (16–18 h), using a suitable inoculum assuming doubling times of 1–1.5 h. The cells were harvested in exponential growth phase at OD_600nm_ = 0.05–0.6 by centrifugation at 14,000 × *g* for 5 min. The pellet was resuspended in fresh BHI supplemented with 1 µg/mL DNA, either genomic DNA of BfR-CA-15687, BfR-CA-14430-strep (35) or methylated 16S rRNA gene fragment. DNA uptake, recombination, and outgrowth of phenotypic resistance were accomplished by incubation for 4 h at 37°C. After incubation, cell suspensions were serially diluted in BHI, plated on ColbA with and without tobramycin or streptomycin and incubated for 48 h at 37°C. The transformation rate was calculated as the ratio of the number of transformants grown on ColbA supplemented with the respective antimicrobial and the total number of colonies on non-selective plates.

### Antimicrobial susceptibility testing using microdilution

The susceptibility testing using broth microdilution method followed the guidelines outlined in M45-A and VET06 (56, 57). Isolates subcultured on ColbA at a temperature of 42°C for 20 ± 2 h were inoculated into cation-supplemented Mueller-Hinton broth (Thermo Fisher Scientific Inc., Waltham, MA, USA) with 5% fetal calf serum (PAN-Biotech, Aidenbach, Germany) (CAMHB/FCS) at a bacterial concentration ranging from 2 to 8 × 10^5^ CFU/mL. The MICs were determined using the European standardized EUCAMP3 plate (Thermo Fisher Scientific Inc., Waltham, MA, USA). In addition, custom plate formats were prepared, incorporating the subsequent antimicrobial agents (Sigma Aldrich, St. Louis, MO, USA) and their concentration ranges depicted in Table 1. Stock solutions of the antimicrobials were dissolved in H_2_O. The U-bottom microtiter plates (Greiner Bio-One International GmbH, Frickenhausen, Germany) were prepared by adding 50 µL of CAMHB/FCS supplemented with the corresponding double-concentrated antimicrobial agent per well. Before use, the sealed plates were stored at 4°C for 24 h. The isolates were prepared following the described method above, with the exception that the inoculum was double concentrated in a volume of 50 µL, which was added to each well of the pre-prepared customized plates. Samples were incubated at 37°C for 48 h. The determination of MICs (in mg/L) was performed using the semi-automated Sensititre Vizion system (Thermo Fisher Scientific Inc., Waltham, MA, USA) and the Sensivizion V2.0 software (MCS Diagnostics BV, Swalmen, The Netherlands). The determination of antimicrobial resistance in *Campylobacter* was based on the guidelines established by the European Committee on Antimicrobial Susceptibility Testing (EUCAST) for Epidemiological cutoff Values (ECOFFs) (58). “Elevated non-wild-type MICs” were previously defined for kanamycin resistance (21). For apramycin and tobramycin “elevated non-wild-type MICs” were defined as >16 mg/L. All ECOFFS and elevated non-wild-type MICs are depicted in Table 1. For quality control, *C. jejuni* strain DSM 4688 and *C. coli* strain 2012-70-443-2 were included, which displayed sensitive phenotypes.

### Assessment of stability of acquired antibiotic resistance

To assess the stability of the acquired resistance determinant, fresh transformant colonies were first serially diluted to form new single colonies on non-selective ColbA plates. Subsequently, representative colonies were repeatedly subcultured on non-selective ColbA at 42°C for 20 ± 2 h. Following the indicated number of passages, bacteria were resuspended in 1 mL buffered peptone water (10 g/L peptone, 5 g/L NaCl, 9 g/L Na_2_HPO_4_ × 12 H_2_O, 1.5 g/L KH_2_PO_4_, pH 7.0 ± 0.2 at 25°C), serially diluted to approximately 200 colonies in 100 µL and plated on ColbA. After incubation at 37°C for 48 h under microaerobic conditions, colonies were transferred by stamping with velvet cloth on a series of ColbA plates containing 4, 8, and 16 mg/L tobramycin and, as the last plate, ColbA without antimicrobial. The orientation of the plates during stamping was carefully marked, ensuring comparison of plate images, which were taken after 48 h of incubation at 37°C using the G:BOX CHEMI XX6 imaging system (Synoptics Ltd, Beacon House, Nuffield Road, Cambridge) controlled by the Genesys V1.8.5.0 software (Synoptics Ltd, Beacon House, Nuffield Road, Cambridge, United Kingdom).

### Whole-genome sequencing

*Campylobacter* isolates were subcultured on ColbA for 20 ± 2 h at 42°C. Bacteria were harvested from 1 mL cells at OD_600nm_ of 2 by centrifugation at 14,000 × *g* for 5 min. DNA extraction for short-read sequencing was performed using either the Maxwell RSC Cultured Cells DNA Kit (Promega Corporation, Fitchburg, WI, USA) or the PureLink Genomic DNA Mini Kit (Thermo Fisher Scientific, Waltham, MA, USA). For long-read sequencing, DNA extraction was performed using either the MagAttract HMW Genomic Extraction Kit (Qiagen N.V., Venlo, The Netherlands) according to the protocol but with a 1.5 h of 56°C incubation step or the MasterPure Complete DNA & RNA Purification Kit (Biozym Scientific GmbH, Hessisch Oldendorf, Germany) following the manufacturer’s protocol but using a more concentrated RNase A solution (100 mg/mL; Qiagen N.V., Venlo, The Netherlands).

The DNA quality was assessed through spectral analysis using a NanoDrop Spectrophotometer (Thermo Fisher Scientific, Waltham, MA, USA), while the concentration was determined using a Qubit 3.0 Fluorometer with the dsDNA BR Assay Kit (4–2,000 ng; Thermo Fisher Scientific, Waltham, MA, USA). DNA libraries for short-read sequencing were prepared using the Illumina DNA Prep, (M) Tagmentation Kit (Illumina, Inc., San Diego, CA, USA), with the modification of using half the volume of all reagents. The Illumina MiSeq benchtop sequencer, equipped with the MiSeq reagent kit v3 (600 cycles, Illumina, Inc., San Diego, CA, USA), or the Illumina NextSeq 500 sequencer utilizing the NextSeq 500/550 mid output kit v2.5 (300 cycles, Illumina, Inc., San Diego, CA, USA) was employed for paired-end sequencing. The read lengths were set from 2 × 149 to 2 × 301, depending on the instrument used.

DNA libraries for long-read sequencing were prepared using the Rapid Barcoding Kit 96 (SQK-RBK110.96, Oxford Nanopore Technologies Limited, Oxford, United Kingdom). The sequencing process was performed on the MinION Mk1C instrument, using either the MinION R9.4.1 or R10.4.1 FlowCell (Oxford Nanopore Technologies Limited, Oxford, United Kingdom). To process the Oxford Nanopore Technology sequencing data, the Guppy basecaller v. 6.4.8 (Oxford Nanopore Technologies, Oxford, United Kingdom) was used in the “super-accuracy” mode.

### Bioinformatic analysis

The Illumina paired-end reads were subjected to trimming and *de novo* assembly using the AQUAMIS pipeline, version 1.3.8 (59). For data quality assessment, reads were considered satisfactory if they exhibited a base accuracy of Q30 (error rate 1:1,000) for over 80% of the reads, and if the minimum read coverage was at least 40. For ONT reads, quality control and assembly were conducted using the MiLongA Pipeline v1.0.1 (60). This pipeline includes various tools, such as porechop v0.2.4 (61) for trimming and Unicycler v0.4.8 (62) for hybrid assembly. Quality was considered sufficient if the filtered median fragment length (*N*_50_ value) was >10,000 and the read coverage exceeded the minimum of 30.

The assembled contigs underwent analysis using the BakCharak pipeline v3.0.4 (63), which incorporates the antimicrobial resistance gene finder module. This module utilizes AMRFinderPlus v3.10.45 (64) and the corresponding AMRFinder database 2023-04-17.1 to identify resistance determinants, applying default thresholds. Furthermore, ResFinder v4.1 (65) was employed on assembled and trimmed read data with lowest thresholds applied (30% identity, 20% coverage) to complement AMRFinderPlus results. Assembled genome contigs were annotated with Bakta (66).

**Fig 5:**
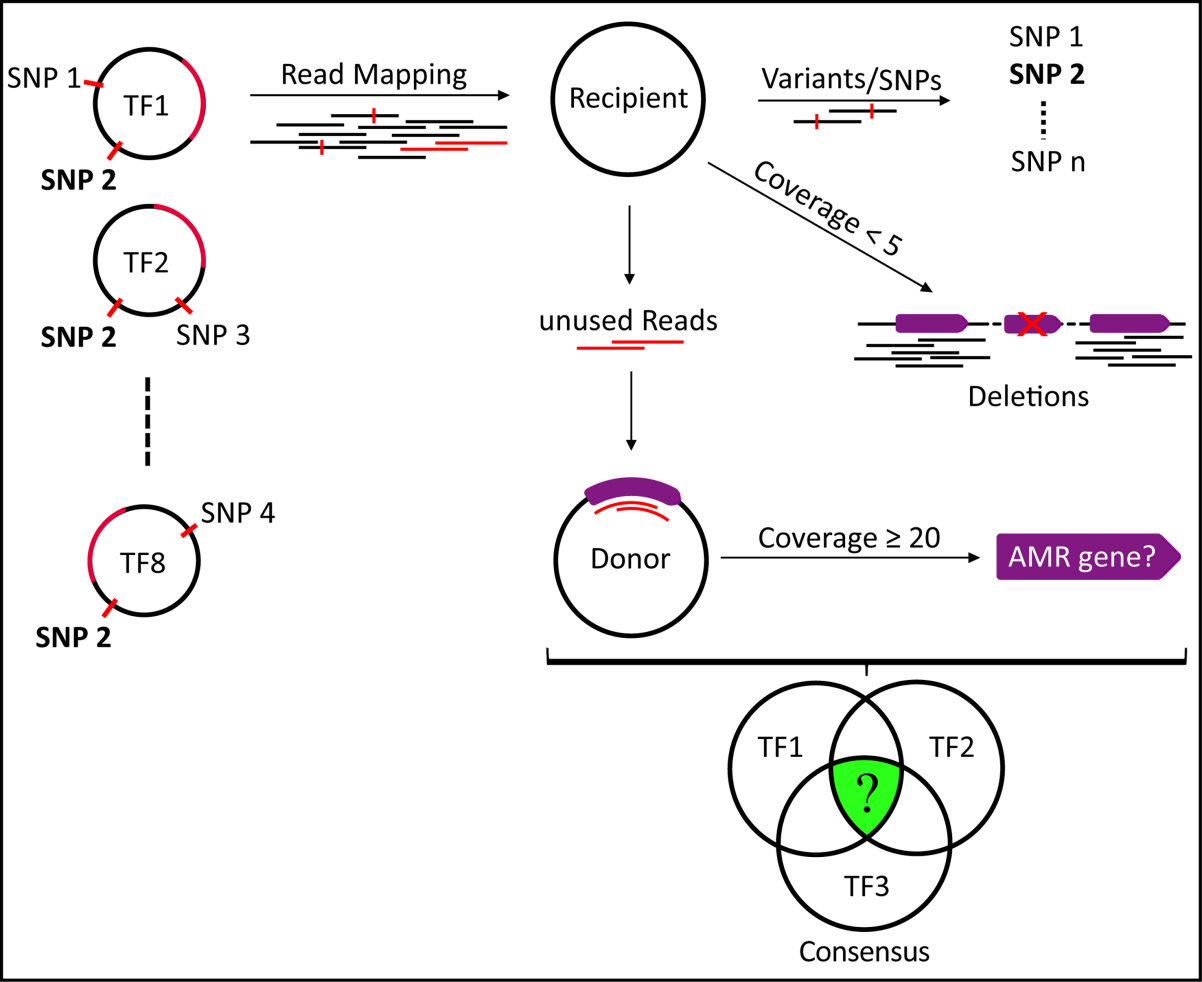
Schematic illustration of the strategy for identification of the novel resistance determinant. Trimmed short-reads from resistant transformants were mapped to the Unicycler hybrid assembly of the recipient. Variants/SNPs and deletions were revealed per transformant. For each transformant, unused reads were subsequently mapped to the Unicycler hybrid assembly of the donor isolate in order to find AMR gene transfer. The consensus of SNPs, deletions, and insertions of all transformants was verified to be present in the donor, but absent in the recipient isolates.

Geneious Prime 2023.2.1 (Biomatters Ltd., New Zealand) was employed to conduct mapping of trimmed reads to assemblies and to analyze sequence variations of the transformants relative to the recipient (Fig. 5). In detail, the trimmed short-reads obtained from the resistant transformants were mapped to the bakta annotated Unicycler hybrid assembly of the recipient isolate. Deletions in the transformants relative to the recipient were identified by displaying low coverage regions (maximum coverage of 5 reads). Variations/SNPs were identified using a minimum coverage of 40 and a minimum variant frequency of 0.8. The paired unused reads were subsequently mapped to the bakta annotated Unicycler hybrid assembly sequences of the donor isolate, which had been concatenated (chromosome and plasmid), to investigate the transfer of larger sequences, such as putative genes or antimicrobial resistance islands [minimum coverage regions (≥20 reads)]. Finally, the consensus of the SNPs was found using the “Compare Annotations” function (Table S1). Putative deletions in common among the various resistant transformants were detected by tracking “low coverage” regions in the recipient (Table S2). Potential insertions were identified by the detection of “high coverage” regions of “unused reads” in the donor, which did not map to the recipient (Table S3). Nucleotide sequences of 16S rRNA genes were aligned using Geneious Prime software with the Geneious Alignment algorithm.

The results retrieved from Sanger sequencing were analyzed with SeqMan Pro 17 (DNASTAR Lasergene 17, DNASTAR, Inc., Madison, WI, USA).

A small fragment (positions 1357–1433) of the 16S rRNA of BfR-CA-15687 comprising the A1387G transition was used to search against NCBIs Nucleotide collection (nr/nt) and whole-genome shotgun contigs (wgs) databases using BLASTN 2.15.0+ (67). Limitations were set for the latter database as *Campylobacter coli* (taxid:195).

## Data Availability

The complete sequence of the BfR-CA-15687 genome (including plasmid) can be found at the National Center for Biotechnology Information (NCBI; genome accession CP126367-CP126368, BioSample accession SAMN34728731). The 16S ribosomal RNA gene sequence of BfR-CA-15687 is available as GenBank accession no. PQ227239.

## SUPPLEMENTAL MATERIAL

Supplemental figures aac.00833-24-s0001.pdf Figures S1 and S2.

Supplemental tables aac.00833-24-s0002.xlsx Tables S1 to S3.

ASM does not own the copyrights to Supplemental Material that may be linked to, or accessed through, an article. The authors have granted ASM a non-exclusive, world-wide license to publish the Supplemental Material files. Please contact the corresponding author directly for reuse.
