# Supplementary material for: The point mutation A1387G in the 16S rRNA gene confers aminoglycoside resistance in Campylobacter jejuni and Campylobacter coli
Source: Antimicrob Agents Chemother. 2024 Oct 15;68(11):e00833-24. doi: 10.1128/aac.00833-24 (PMC11539217; doi:10.1128/aac.00833-24)
Supplement: Supplemental figures — Figures S1 and S2. [file aac.00833-24-s0001.pdf]

Supplementary Figures

The point mutation A1387G in the 16S rRNA gene confers aminoglycoside resistance in *Campylobacter jejuni* and *Campylobacter coli*

Michael Zarske, Christiane Werckenthin, Julia Golz, Kerstin Stingl

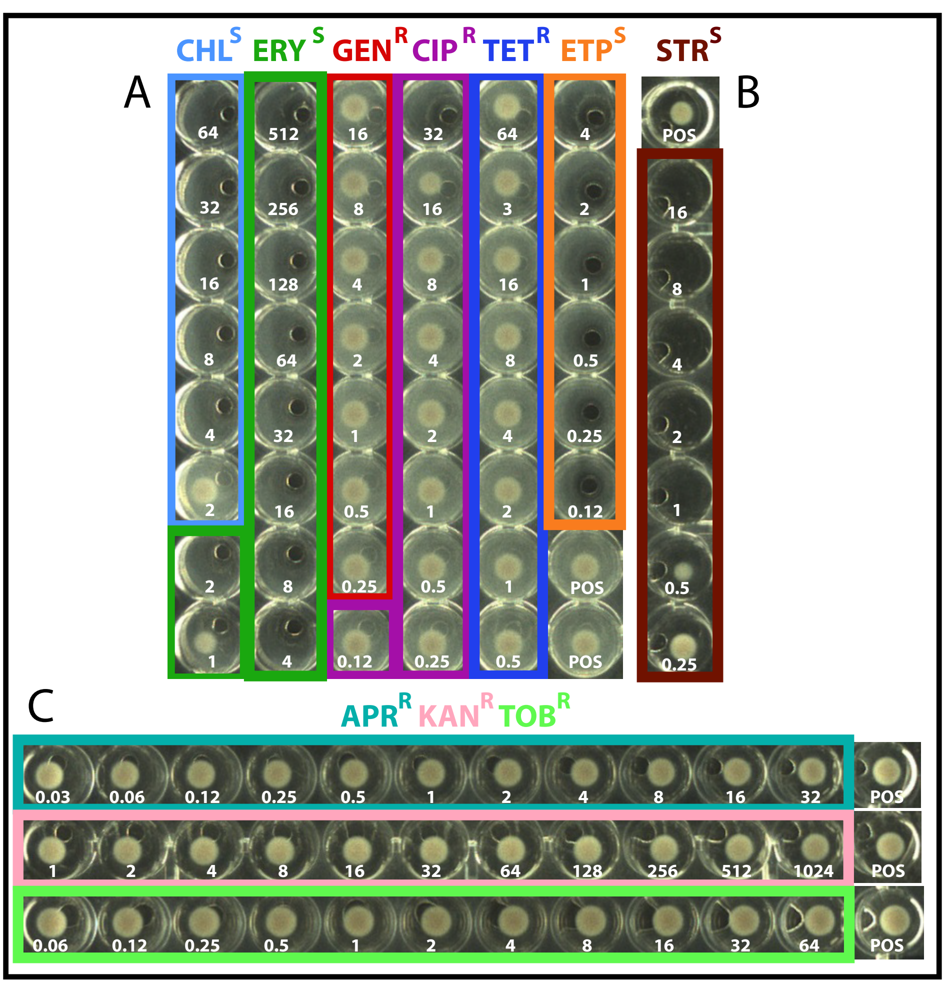

Figure S1. Antimicrobial susceptibility testing performed on *C. coli* BfR-CA-15687 using the standardized EUCAMP3 (A) and custom plates (B, C). The isolate exhibited resistance to CIP, GEN and TET and was sensitive to CHL, ERY, ETP and STR (A, B). Moreover, elevated non-wildtype MIC values were observed for APR, KAN and TOB (C). Test concentrations in mg/L are indicated by white numbers within each well; colored frames indicate the corresponding wells of the respective test substance. POS, positive control without antimicrobial substance; APR, apramycin; CHL, chloramphenicol; CIP, ciprofloxacin; ERY, erythromycin; ETP, ertapenem; GEN, gentamicin; KAN, kanamycin; TET, tetracycline; TOB, tobramycin; STR, streptomycin.

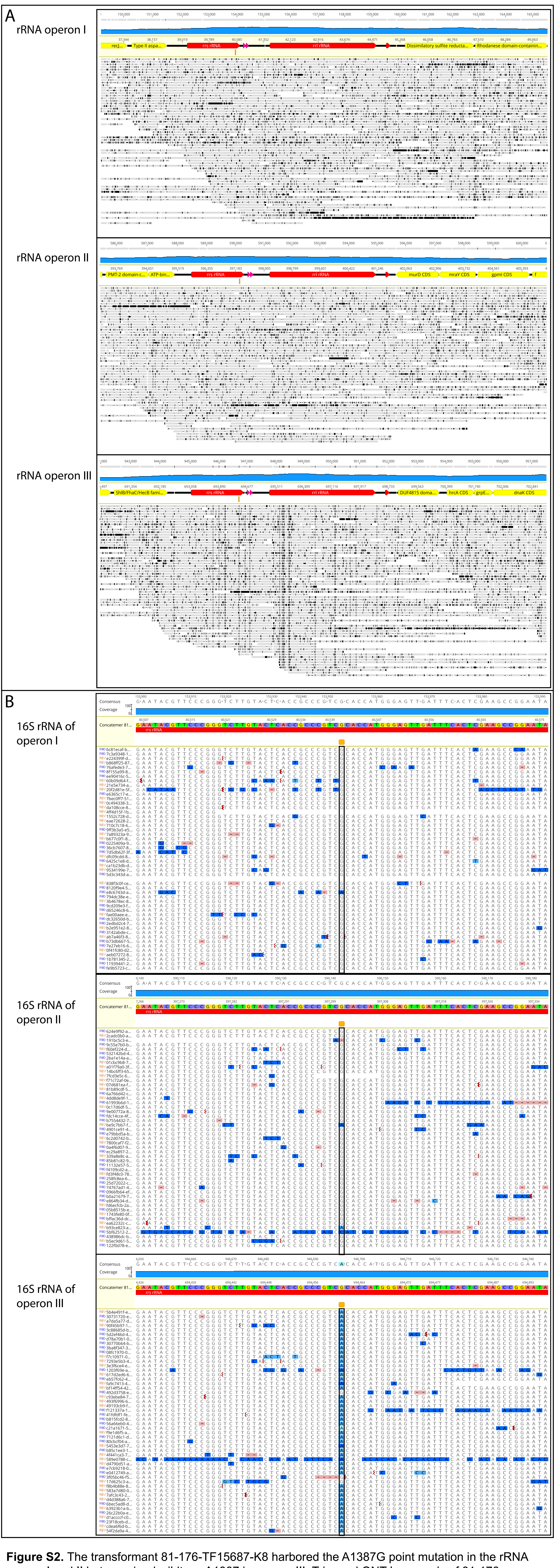

Figure S2. The transformant 81-176-TF15687-K8 harbored the A1387G point mutation in the rRNA operon I and II but remained wildtype A1387 in operon III. Trimmed ONT long reads of 81-176-TF15687-K8 were mapped to its unicycler hybrid assembly. Overview of the different gene contexts of rRNA operon I-III confirmed that long reads covered 5' and 3' regions specifically flanking the respective rRNA operon (A). The sequence sourrounding position 1387 (highlighted with a black frame) in the respective 16S rRNA genes is depicted in (B). Red arrows, rRNA genes; yellow arrows, genes flanking the rRNA operons; grey bars, trimmed long reads mapped to the hybrid assembly sequence (in black (A) or blue/red (B) missmatches/insertions/deletions relative to the hybrid assembly; blue bar, coverage of mapped reads. Ruler indicates location in the hybrid assembly genome.
